# Neural responsiveness to Chinese versus Western food images: An functional magnetic resonance imaging study of Chinese young adults

**DOI:** 10.3389/fnut.2022.948039

**Published:** 2022-08-12

**Authors:** Xi Xu, Jiajia Pu, Amy Shaw, Todd Jackson

**Affiliations:** ^1^School of Psychology, Southwest University, Chongqing, China; ^2^Department of Psychology, University of Macau, Macao, Macao SAR, China

**Keywords:** food images, neural activation, fMRI, culture, food preferences, Chinese

## Abstract

Cross-cultural studies suggest that people typically prefer to eat familiar foods from their own culture rather than foreign foods from other cultures. On this basis, it is plausible that neural responsiveness elicited by palatable food images from one’s own culture differ from those elicited by food depictions from other cultures. Toward clarifying this issue, we examined neural activation and self-report responses to indigenous (Chinese) versus Western food images among young Chinese adults. Participants (33 women, 33 men) viewed Chinese food, Western food and furniture control images during a functional magnetic resonance imaging (fMRI) scan and then rated the images on “liking,” “wanting,” and “difficult resisting.” Analyses indicated there were no significant differences in self-report ratings of Chinese versus Western food images. However, Chinese food images elicited stronger activation in regions linked to cravings, taste perception, attention, reward, and visual processing (i.e., cerebellum crus, superior temporal gyrus, supramarginal gyrus, middle temporal gyrus, inferior parietal lobule, posterior insula, middle occipital gyrus; inferior occipital gyrus). Conversely, Western food images elicited stronger activation in areas involved in visual object recognition and visual processing (inferior temporal gyrus, middle occipital gyrus, calcarine). These findings underscored culture as a potentially important influence on neural responses to visual food cues and raised concerns about the ecological validity of using “standard” Western food images in neuroimaging studies of non-Western samples. Results also provide foundations for designing culturally informed research and intervention approaches in non-Westerns contexts guided by the use of external food cues that are most salient to the cultural group under study.

## Introduction

Cross-cultural studies have indicated that people typically show stronger overall preferences for foods that are familiar to their own cultural group than less familiar, foreign foods (1, 2). Consequently, neural responses elicited during exposure to images of foods from one’s local culture may also differ from those elicited during exposure to foods imported from other cultures. As such, the implicit assumption that “standardized” images of palatable Western foods are appropriate for use in neuroimaging studies of responses to food cues in non-Western groups warrants scrutiny. Toward testing this premise, we examined neural responses and subjective reactions of mainland Chinese young adults during exposure to images of traditional Chinese foods versus Western foods more typically depicted in neuroimaging studies of non-Western samples.

Taste is a critical factor shaping food preferences (3, 4). For example, studies of adolescents in the United States (5) and non-Western nations including China (6) indicate taste is the most important influence on food selection. Neophobia, the tendency to reject or avoid novel edibles while developing preferences for more familiar foods (3), is an important genetic predisposition that shapes preferences for particular foods (4). While neophobic tendencies are encoded in genetics, their expression is shaped by culture and can differ between countries (7, 8). Past studies have found that unfamiliar taste and appearance deter people from trying ethnic food (9) while familiarity influences food preferences and willingness to eat novel foods across cultures and socio-demographic groups (3, 10, 11).

As an illustrative example, despite considerable diversity in food preferences between its different regions, traditional culture and food tastes in China have strong roots (12). Based on Chinese traditions, good food should be excellent in terms of color, aroma and taste (2). “Proper meals” in Chinese culture comprise appropriate amounts of starches (e.g., rice, noodles), vegetables and meats, and lower amounts of dairy products and sweets (13). As well, flavors that distinguish Chinese cuisine include a soy sauce, rice wine, and ginger mixture (14). This traditional pattern provides less total fat, saturated fat, cholesterol, and calcium than the typical American dietary pattern does (13).

Studies of Chinese samples suggest food preferences are affected by culture (1, 13–17). For example, among residents of Shanghai and Xi’an, “unfamiliar,” “sweet,” and “greasy” were unique, negatively valenced taste descriptors of European foods while “safe” and “upscale” were endorsed as positively valenced descriptors (15). In another China-based study of adolescents, the most frequently consumed snacks included fruit, milk and instant noodles (16); underscoring possible aversions to sweet foods, these snacks were consumed at least 2–3 times more often than soft drinks, candy/gum and chocolate. Among Asian travelers to Australia, Chang et al. (14) found Chinese food was highly preferred despite immersion within a new culture. Even when participants were eager to try local delicacies, they sought Chinese foods during mealtimes because the “acceptability” and “palatability” of these dishes were guaranteed and mitigated shock experienced from eating less familiar foods. Many also sought “familiar flavors” in local Australian food and welcomed the fusion of Chinese and Australian food to foster acceptance of unfamiliar foods. Finally, in contrast to “palatability,” motives for trying Australian foods included a desire to learn about the culture, increase Western culinary knowledge, develop memories about Australian holiday experiences, and assert prestige and status. Similarly, Chinese travelers to Spain preferred eating Chinese food and familiar fast food relative to unfamiliar local Spanish food (17). Finally, Li (1) found that although Chinese cruise tourists traveling abroad were initially willing to try novel Western foods, they were subsequently more reluctant due to being less accustomed to the taste, type, preparation and temperature of Western foods; these reactions were especially strong among older adults who became neophobic to Western food.

Notwithstanding evidence that Chinese samples prefer familiar Chinese foods in contrast to less familiar Western foods, concerns have emerged about diets becoming increasingly Westernized in China (18, 19). Wang et al. (19) noted the remarkable growth of U.S.-based fast-food restaurants in China during the past 30 years with McDonald’s franchises expanding at a rate of nearly 10 new restaurants per week due to their perceived convenience, safety, and prestige. Given the proliferation of Western food products in China, it is also possible that reactions to Western foods do not diverge sharply from those of indigenous Chinese foods, particularly among children, adolescents, and young adults to whom Westernized diets are marketed.

Despite increased research in non-Western contexts such as China, studies on food consumption and food representations typically have comprised North American and European samples (20). Augmenting dominant North American and European perspectives on these issues with data from Asian contexts has the potential to elucidate how cultural background informs preferences for and brain responses to presentations of foods from local and non-local cultures.

In sum, numerous China-based self-report studies have underscored stronger taste preferences for familiar Chinese foods as opposed to novel foods from other cultures, albeit conflicting data highlight increasingly Westernized diets in China, especially among younger cohorts. Functional magnetic resonance imaging (fMRI) has been essential in documenting underlying neural activation patterns elicited by images of palatable foods. Though not exhaustive, food image presentations can elicit increased activity in reward/craving circuitry regions including the anterior and posterior insula (PI), orbital frontal cortex (OFC), anterior cingulate cortex (ACC), caudate, hippocampus, amygdala, and dorsolateral prefrontal cortex (DLPFC) (21–25) as well as regions involved in taste perception, olfaction, temperature, and texture perception (e.g., insula/operculum, left OFC, pregenual cingulate cortex) (26–28). Exposure to food images can also elicit activation of regions linked to visual processing (e.g., fusiform gyrus, occipital gyrus, superior parietal lobule, cuneus) (26–28) and attention/cognitive control (e.g., precuneus, inferior parietal lobe, middle frontal gyrus) (26, 29, 30).

A majority of related fMRI studies has employed images of palatable Western foods in samples from the United States or Europe (21, 26, 31–35). Similarly, research on Chinese samples (36–38) has tended to employ standard Western food images even though these may be less preferred and/or less frequently consumed than food staples typical of Chinese diets are. Consequently, it is plausible that neural activity patterns elicited during exposure to palatable Western food images differ from patterns elicited during exposure to palatable indigenous food images that represent daily diets and/or food preferences of most mainland Chinese residents.

To test this premise, we evaluated self-reported reactions to and neural responses elicited by images of indigenous (i.e., local Chinese) versus Western food dishes among mainland Chinese young adults. Following from behavior studies of food preferences in Chinese samples reviewed above, we hypothesized that Chinese food images would elicit higher ratings of liking, wanting and difficulty resisting than Western food images would. Based on the assumption that subjective preferences for Chinese (versus Western) foods might reflect underlying differences in neural activation, we also tested an exploratory hypothesis that Chinese food images would elicit comparatively stronger neural activation responses than Western food images would in regions linked to reward/cravings circuitry, taste perception, attention and inhibitory control.

## Materials and methods

### Participants

The initial sample comprised 67 students (34 women, 33 men) recruited from a major university in China. On average, participants were 20.05 years of age (*SD* = 1.62, range: 18–25 years) and had an average body mass index (kg/m^2^) of 25.41 (*SD* = 5.65, range: 18.12–40.14). Exclusion criteria included the presence or history of major medical conditions or psychiatric illnesses including clinical eating disorders, previous or current prescription medications, and significant visual impairments. Specific contraindications for fMRI (i.e., pacemaker, metal implants, dentures, severe claustrophobia) were other exclusion criteria.

### Image sets

**FIGURE 1:**
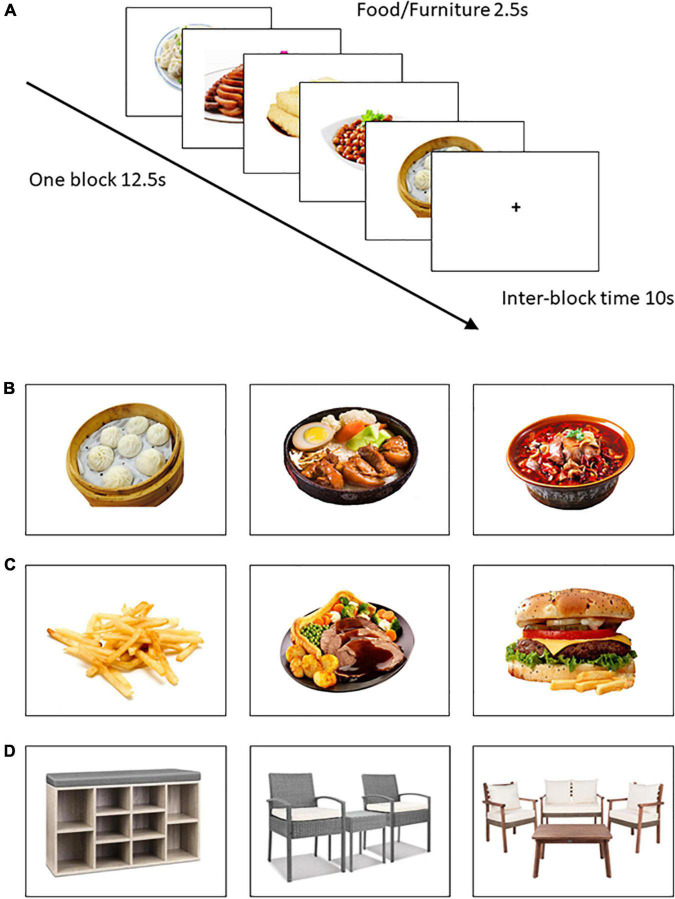
Examples of image types and the sequence of events in each task trial. **(A)** Depicted is one Chinese food block; **(B)** Chinese food (ChFd); **(C)** Western food (WeFd); **(D)** Furniture (Furn).

Images were 180 color photographs representing three image categories: 60 Chinese food items (e.g., dumplings, noodles, tofu), 60 Western food items (e.g., hamburger, French fries) and 60 furniture items (e.g., tables, cabinet, chairs) that served as control images (Figure 1). Chinese and Western food images were selected from the Food Image Library of the Psychology Department of the affiliated university (39). Some Western food images were selected from another food image database (40). Furniture images were obtained from freely available websites. For standardization, all images of Chinese food, Western food, and furniture were displayed in same resolution (720 × 540 pixels) and featured against a white background (41).

The final image set was selected from a pilot study in which 10 undergraduates (five women, five men), who did not participate in this study, sorted images into the three categories listed above. Participants were provided with definitions and sample images of each image category prior to the sorting task. During the task, a question was displayed below the image querying, “What kind of image do you think this is?” with A = Chinese food item, B = Western food item, and C = furniture item as the choices. Only images correctly classified within their appropriate category at rates of 90% or higher were retained in the final image set. Highlighting equivalent levels of familiarity and distinctiveness between these image sets, mean classification accuracy rates for retained Chinese food images (98%), Western food images (100%), and furniture images (100%) were near perfect and far exceeded chance alone rates (33%). In addition, rater judgments of image visual complexity did not differ between the three image categories, *F*(2,177) = 0.513, *p* = 0.600. Finally, although calorie counts of individual food images varied, Chinese versus Western food image sets did not differ significantly in terms of (i) average calories based on the operationalization of high calorie foods as those with an energy density ≥ 1.5 kcal/gram (42, 43) and calorie information from related databases (40, 44), percentages of high calorie food image [92% versus 85%, χ^2^(1, *N* = 120) = 1.29, *p* = 0.26], (ii) overall food content (e.g., meats, vegetables, etc.), χ^2^(2, *N* = 66) = 1.31, *p* = 0.52, based on operationalizations from other published sources (45), (iii) how food was presented (alone or in white dish against white background versus in non-white dish/container against white background), χ^2^(1, *N* = 120) = 2.83, *p* = 0.093, or (iv) flavor (i.e., sweet, savory, blandness), χ^2^ (2, *N* = 120) = 2.89, *p* = 0.39.

### Procedure

The study was approved by the Human Research Ethics Committee of the affiliated university. An *a priori* power analysis with the G*Power Version 3.1 (46) was used to estimate sample size based on a repeated measures analysis of variance (ANOVA) with the following parameters: a medium effect size (Cohen’s *f*) = 0.35, alpha = 0.05, power (1-β) = 0.95. The analysis resulted in an estimated sample N of 64, though we recruited beyond this level anticipating a 5–10% attrition rate.

Participants were solicited *via* an advertisement on the university’s electronic bulletin board seeking volunteers for a study on reactions to different kinds of images. Prospective volunteers completed an online screen that included demographics (age, gender, height, weight), exclusion criteria and MRI contraindications. Appointments were made with eligible, willing volunteers. They were asked to wear light, comfortable clothing and consume their regular meals but refrain from eating or drinking (except water) for at least 2 h before their appointment (47) to better ensure they were not overly hungry or satiated during testing. Upon arrival, participants removed their shoes and objective measures of height and weight were taken to calculate body mass index (BMI). Next, measures of demographics (age, gender, Han versus minority ethnicity), parental education (high school completion or lower versus more than high school completion) were completed with single item ratings of current hunger and fullness anchored by 0 (not at all) 50 (somewhat), and 100 (extremely) (38). Time since last meal was assessed with the query, *“How long has it been since your last meal (in minutes*)?”

Prior to scanning, participants were instructed to lie still, remain awake but relaxed, think of nothing in particular while keeping their eyes open, and focus carefully on each image presented to them. At the scan outset, written instructions were presented: “In the next task you will see food and non-food images. Please pay close attention to the images since you will be tested on their nature at the end of the MRI session.” The scan then proceeded with each trial comprising one full-color image presented once for 2.5 s. Participants viewed food/control images through an adjustable mirror mounted on the scanner head coil. Following other published work (48), each block comprised five images of the same type (Chinese food, Western food, or furniture) presented with no pauses (see Figure 1). Blocks were separated by a 10 s inter-block interval featuring a central fixation cross. The total run comprised 36 blocks (820 s) including a 30 s rest after 18 blocks. Blocks were presented pseudo-randomly so that the same image type was never presented more than twice in a row. Immediately after the fMRI run, participants were asked if they had fallen asleep during the scan; none reported having done so. They also completed a simple memory item in which they were asked to identify two image categories from the following list that were NOT presented in the immediately preceding viewing task: cars, Chinese foods, people, Western foods, and furniture. Data from one female participant who answered this item incorrectly were excluded due to potential inattention.

After leaving the scanner, participants rated each image on computer in a separate room. Following other published research (49), respondents rated how much they (1) liked each portrayed image (anchored by *1* = *dislike extremely and 5* = *like extremely*), (2) wanted to eat (or use) each depicted stimulus (anchored by *0* = *do not at all, 50* = *want somewhat, and 100* = *definitely want*), and (3) had difficulty to resist eating or using each depicted stimulus (anchored by *0* = *definitely not difficult, 50* = *somewhat difficult, 100* = *definitely difficult*). For wanting and difficulty resisting ratings, any number between 0 and 100 could be selected. Images were presented in random order to control for potential order effects or participant “response sets.” After the rating task, participants were asked to guess the main study hypotheses, debriefed, and paid 100 RMB for their time and effort. On average, this study took 35–45 min to complete.

### Functional magnetic resonance imaging data acquisition and preprocessing

Images were acquired *via* a Magnatom Terra 7T MRI (Siemens Medical, Erlangen, Germany) with a 64-channel head coil. Soft foam pads were used to decrease head motion and scanner noise. Image presentations and self-report ratings were controlled by a computer outside scanner using E-prime 2.0 (50). Functional images were collected using a T1-weighted gradient echo-planar imaging (EPI) sequence [repetition time (TR) = 2000 ms; echo time (TE) = 30 ms; Flip Angle = 90°; voxel size = 2 mm × 2 mm × 2.3 mm; Field of View (FoV: AP, FH, RL) = 224 mm × 143 mm × 224 mm, slices = 62]; 410 BOLD images were collected. A high-resolution T1-weighted anatomical image was acquired for precise normalization, using a standard MPRAGE (magnetization prepared rapid acquisition gradient echo) sequence [repetition time (TR) = 2530 ms; echo time (TE) = 2.98 ms; Field of View (FoV: AP, FH, RL) = 224 × 256 × 192 mm^3^; voxel size = 0.5 × 0.5 × 1 mm^3^; slices = 192].

Data were preprocessed using DPABI (Data Processing & Analysis for Brain Imaging) (51) in MATLAB (version 2014a, Math Works, Natick, MA, United States). Both functional images and T1 images were converted from DICOM to NIFTI format. Slice timing was conducted to correct slice order, functional images were realigned to the first volumes, and six head-motion parameters were estimated from three translation and three rotation vectors. All images were skull stripped. T1-weighted images were co-registered to averaged functional images and segmented into constituent tissues using a DARTEL template (52). Functional images were normalized to the MNI (Montreal Neurological Institute) space with the DARTEL template. Spatially normalized echo-planar images were smoothed with Gaussian kernel of 8 × 8 × 8 mm FWHM (Full-width-at-half-maximum) (53).

### Data analysis

#### Self-report data analyses

Descriptive statistics summarized sample characteristics, state hunger/fullness ratings, and reactions to the three image types. Repeated measures analyses of variance (ANOVAs) assessed within sample differences in ratings of (1) liking, (2) wanting, and (3) difficulty resisting Chinese food versus Western food versus control (furniture) images. Least significant difference (LSD) *post hoc* tests were run to identify specific image type differences when ANOVA values were significant. These analyses were conducted using SPSS 26 (54) with *p* < 0.05 significance cutoffs. Finally, Spearman correlation coefficients were calculated to examine intercorrelations between BMI and self-report ratings of hunger, fullness, time since last meal, image liking, wanting, and difficulty resisting ratings.

#### Imaging data analyses

Image preprocessing and multiple corrections were performed with DPABI. We used SPM12 (Statistical Parametric Mapping), run on MATLAB 2021, for analyses and Bspmview (55) for results presentations. In first-level analyses, task-related activity within each participant’s data was detected by convolving the canonical hemodynamic response function (HRF) with a boxcar function representing onsets and durations of different conditions. Image type conditions were treated as regressors and head motion parameters were treated covariates in a generalized linear model (GLM). For the GLM analysis, Chinese food, Western food, and furniture image type conditions were generated to model the boxcar function *via* a sustained epoch representing each stimulus duration. Main analyses featured within sample contrasts between Chinese vs. Western food images. Supplementary analyses included within sample contrasts between Chinese or Western food images vs. furniture.

In second level analyses, at a group level, contrasts from individual participants were first entered into one-sample *t*-tests to measure brain response differences regarding the within sample contrasts. Scan time served as a covariate. A family-wise error (FWE) correction based on Gaussian random field (GRF) theory was applied to control for false positives resulting from multiple comparisons at the α = 0.05 level (56) and cluster-based inferences about findings for which there were likely to be significant activation effects (45, 57). The corrected height-extent threshold was calculated for numbers of voxels (k) in each statistical map across the whole brain. To identify significant activations for Chinese food vs. Western food, Chinese food vs. furniture and Western food vs. furniture contrasts, a *p* < 0.001 threshold was submitted to cluster detection within DPABI; cluster sizes of 178 voxels, 222 voxels and 231 voxels, respectively, resulted in corrected probabilities of *p* < 0.05. Finally, Spearman correlation coefficients were calculated to examine relations of BOLD signals in clusters from the Chinese versus Western food image contrast that included structures previously implicated as “reward” regions with measures of BMI, hunger, fullness, time since last meal, image liking, wanting, and difficulty resisting ratings. We extracted beta values in BOLD signals of these clusters using Marsbar (58) (sphere radius at 10 mm) and SPM12.

## Results

### Preliminary analyses

Data from one male participant whose hunger rating was nearly three standard deviations above the mean were retained in main analyses because results were very similar to those in which his data were dropped.

### Description of sample characteristics and differences in reactions to image categories

Descriptive statistics for sample demographics and background measures are summarized in Table 1. The sample had significantly higher liking, wanting and difficulty resisting ratings for both Chinese and Western food images than furniture images. However, contrary to predictions, none of these ratings differed for Chinese versus Western food images (see Table 2). Finally, correlation analyses indicated BMI was not related to self-reported hunger or fullness, time since last meal, or ratings of liking, wanting, or difficulty resisting either food image set (all *r*’s < 0.172, all *p*’s > 0.168).

**TABLE 1:** Demographic and background characteristics of sample (*N* = 66).

Measure	%/M ± SD
Gender (Female)	51.5%
Age	20.05 ± 1.66
Body Mass Index	25.21 ± 5.44
Ethnicity (Han majority)	90.9%
**Parent Educational level (≥high school)**	
Maternal	53.0%
Paternal	63.6%
Current Hunger	29.32 ± 24.13
Current Fullness	48.53 ± 21.72
Time since last meal (in minutes)	191.61 ± 155.01

**TABLE 2:** Sample differences in liking, wanting, and difficulty resisting food and furniture image sets (*N* = 66).

Reaction measure	Image type				
	Chinese food (ChFd)	Western food (WeFd)	Furniture (Furn)	*F*	*Post hoc* tests
Liking	3.57 ± 0.47	3.55 ± 0.52	2.91 ± 0.68	41.58***	ChFd > Furn***, WeFd > Furn***
Wanting	51.11 ± 16.81	51.63 ± 17.46	37.44 ± 17.47	41.18***	ChFd > Furn***, WeFd > Furn***
Difficulty resisting	40.18 ± 19.58	41.07 ± 20.49	26.61 ± 18.59	48.44***	ChFd > Furn***, WeFd > Furn***

***p < 0.001.

### Neural activation differences between image categories

#### Activation differences between Chinese food versus Western food images

**FIGURE 2:**
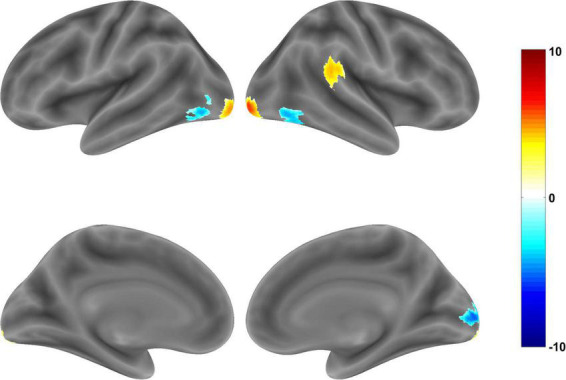
Brain regions showing significant activation in whole brain analysis of Chinese food vs. Western food images (corrected *p* < 0.05). Warm tones indicate greater activation during exposure to Chinese food images and cool tones indicate greater activation during exposure to Western food images.

In the Chinese-Western food image contrast, Chinese food images elicited significantly stronger activation in clusters comprising (i) the right cerebellum crus, (ii) inferior occipital gyrus (IOG) and middle occipital gyrus (MOG), (iii), MOG and (iv) superior temporal gyrus (STG), supramarginal gyrus (SMG), middle temporal gyrus (MTG), inferior parietal lobule IPL, and PI (Table 3). PI involvement was modest based on the DPABI report (14 voxels) and SPM xjView report (20 voxels) but less evident in the anatomical map (Figure 2). Conversely, Western food images elicited significantly stronger activation in visual processing regions including two inferior temporal gyrus (ITG)/MOG clusters, a fusiform gyrus cluster, and a calcarine/MOG cluster (Table 3 and Figure 2).

**TABLE 3:** Activation differences during exposure to Chinese vs. Western food images (*N* = 66).

Contrast	Anatomical label	BA	Hem	Voxels	*x*	*y*	*z*	Peak *T*-value
ChFd > WeFd	Cerebellum crus	–	R	502	42	–78	–41.4	5.39
			R		32	–60	–37	4.23
			R		22	–84	–48	3.86
	MOG/IOG	18/17	L	457	–26	–100	–9.2	5.20
	IOG	18/17	R	449	28	–100	–4.6	6.42
	STG/SMG/MTG/IPL/PI	22/13	R	293	60	–40	20.7	4.65
WeFd > ChFd	ITG/MOG	19/37	L	440	–52	–78	–6.9	5.23
	FFG	37	L		–34	–60	–2.3	4.73
	ITG/MOG	19/37	R	304	48	–70	–9.2	4.28
	Calcarine/MOG	18/17	R	234	10	–96	4.6	6.05

BA, Brodmann area; Hem, Hemisphere; x,y,z coordinates in MNI space (Montreal Neurological Institute); ChFd, Chinese food; WeFd, Western food; L and R, left and right; Reported brain activation was significant at corrected *p* < 0.05; MOG, middle occipital gyrus; IOG, inferior occipital gyrus; STG, superior temporal gyrus; SMG, supramarginal gyrus; MTG, middle temporal gyrus; IPL, inferior parietal lobule; PI, posterior insula; ITG, inferior temporal gyrus; FFG, fusiform gyrus.

#### Associations of self-report measures and image ratings with reward region activation in Chinese versus Western food image contrast

**FIGURE 3:**
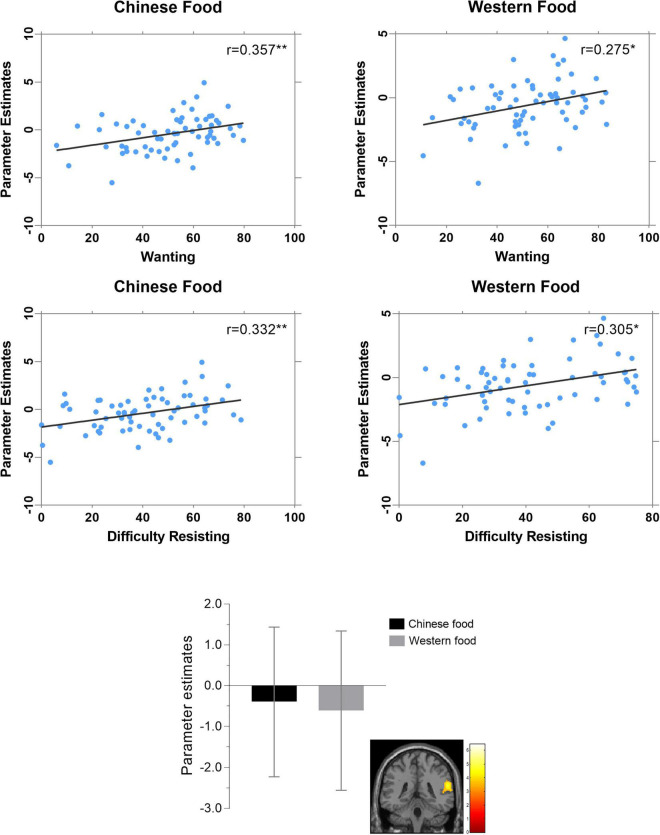
Correlations of food image ratings with BOLD responses of “reward” region cluster (superior temporal gyrus, supramarginal gyrus, middle temporal gyrus, inferior parietal lobule, posterior insula) differentiating Chinese versus Western food images (*p < 0.05, **p < 0.01).

Analyses examining correlates of reward region activation in the Chinese versus Western food contrast indicated responsivity of the STG/SMG/MTG/IPL/PI cluster) was not related to BMI, state hunger or fullness, time since last meal or liking of Chinese or Western food images (Table 4). However, pronounced activation of this cluster had significant positive associations with reported wanting and difficulty resisting Western food images and, especially, Chinese food images (Figure 3). Differences in strengths of relation between right PI-Chinese food versus right PI-Western food image responsiveness on measures of wanting (*r* = 0.36 versus *r* = 0.28) and difficulty resisting (*r* = 0.33 versus *r* = 0.31) were not statistically significant (*p*’s > 0.30).

**TABLE 4:** Bivariate associations between self-report ratings and reward region cluster^1^ differentiating Chinese versus Western food images.

Measure	Food image type	
	Chinese	Western
Body Mass Index	0.10	0.06
Current Hunger	0.06	0.05
Current Fullness	–0.17	–0.13
Time Since Last Meal	0.05	0.04
Liking of Depicted Food	0.19	0.18
Wanting of Depicted Food	0.36**	0.28*
Difficulty Resisting Depicted Food	0.33**	0.31*

*p < 0.05, **p < 0.01 (two-tailed).

^1^Cluster comprising superior temporal gyrus, supramarginal gyrus, middle temporal gyrus, inferior parietal lobule, posterior insula.

#### Supplementary analyses of neural activation differences between food versus furniture image categories

Although no hypotheses were generated, Supplementary Table 1 summarizes activation results based on contrasts of Chinese food-furniture and Western food-furniture contrasts. Chinese food images elicited comparatively stronger activation in the left middle occipital gyrus, calcarine, and bilateral lingual gyrus and comparatively weaker activity in the right calcarine, left precuneus and cerebellum. Western food images elicited relatively increased responsivity in the lingual gyrus, cerebellum, and superior orbital frontal gyrus, and comparatively attenuated activity in the fusiform gyrus, precuneus, calcarine, cerebellum, frontal pole, middle frontal gyrus and angular gyrus (Supplementary Table 1 and Supplementary Figure 1).

## Discussion

This study is the first to evaluate differences in neural activation elicited by and judgments of Chinese versus Western food images among young Chinese adults. The hypothesis that respondents would display comparatively stronger activation in regions associated with reward, gustatory responses, attention, and cognitive control during exposure to Chinese food images received partial support. Conversely, the hypothesis that participants would report corresponding elevations in liking, wanting, and/or difficulty resisting Chinese food images was not supported. Potential implications are elaborated in the context of related research on responses to visual food cues.

### Neural activation in response to Chinese versus Western food images

Significantly stronger neural responsivity to Chinese than Western food images was found in select regions related to cravings, taste perception, and attention including the cerebellum crus, STG, SMG, MTG, IPL, and PI. Although the cerebellum is frequently overlooked in studies of neural responses to food images (59), its involvement in higher order functions such as reward-based learning, attention, emotion and executive functions (60) as well as the regulation of eating behaviors (61, 62) and olfaction (63) are widely documented. Some of this literature has underscored cerebellum involvement in processes underlying addiction including reward, motivational drive, salience, and inhibitory control (64). Briefly, the cerebellum crus is related to functional resting state networks and cerebral structures involved in executive control, drug craving, response selection and salience (65, 66). The cerebellum also modulates reward and saliency responses based on reciprocal connections with dopaminergic systems in basal ganglia (64). Previously, fMRI studies on addiction have found significantly increased cerebellum crus activation in response to images of drug use versus neutral images among recently abstinent heroin users (67, 68). Similarly, recently abstinent alcoholics have shown stronger cerebellum crus I activation during exposure to ethanol odors compared to non-patient controls (69). Although we do not claim that heightened cerebellum crus I responsiveness to Chinese versus Western food images in this study illustrates responses to addictive substances, the addiction metaphor has utility in framing fMRI results on responses to food cues (21, 70, 71). As such, our cerebellum crus I results may reflect greater intake of, stronger approach motivation toward, and/or increased automatic preferences for Chinese food images.

A cluster of structures (STG, SMG, MTG, IPL, PI) also showed significantly stronger activation during exposure to Chinese (versus Western food cues); associated functions of these structures include taste perception, reward processing, visual perception, cravings and inhibitory control. The STG is functionally related to the primary gustatory cortex (34). Links between STG responsiveness and taste perception have been found in various studies. For example, significantly stronger STG activation has been observed during presentations of food (versus non-food) images or food (versus non-food) commercials (72–74). Our results diverged somewhat from past studies (73, 74) in that we found no food versus non-food activation differences in the STG. Instead, stronger activation observed during exposure to Chinese (versus Western) food images suggested that differential STG responses are not a reflection of exposure to food *per se* but are due, instead, to indigenous versus non-indigenous culture of food images and related patterns of consumption. Aside from its involvement in taste perception, possibly due to accompanying emotional valences, the SMG has been linked to food reward processing, inhibitory control and eating behavior (59, 75–77). For example, stronger SMG disinhibition corresponds to increased food reward-related brain activation and emotional eating (75). Furthermore, compared with a satiated state, hunger elicits higher SMG functional connectivity with midbrain and limbic regions (59, 76). Increased SMG connectivity with the dorsal anterior cingulate cortex, insula, cerebellum has also been observed among binge-eaters in response to high (vs. low) calorie food cues (77). In the context of these findings, increased SMG activity during exposure to Chinese (versus Western) food images could reflect enhanced reward responsivity, reduced cognitive control, and/or increased motivation to eat.

Regarding other structures involved in this cluster, comparatively stronger activation of the IPL and MTG were also evident in response to Chinese food images. The IPL is a core attentional network node (26, 49) previously found to have enhanced responsiveness to food (versus non-food) images (78) as well as cues for addictions including gaming and smoking (79). In a recent China-based study, extreme cravers of spicy foods, showed significantly stronger activation to spicy than non-spicy food images compared to non-cravers in several regions including both the bilateral insula and right IPL. Furthermore, among extreme cravers, right IPL activation and frequency of spicy food intake had a significant positive correlation, independent of liking (80). In the context of these findings, IPL activity differences from our sample may have reflected increased attention allocation to Chinese food images and/or, more speculatively, higher intake of Chinese foods than Western foods. Differential MTG involvement may reflect increased visual perception or episodic memory elicited by Chinese food images, though one recent study reported increased MTG activation during processing of appetitive or high-calorie food cues (81).

Finally, Chinese (versus Western) food images elicited stronger activation in the PI, albeit involvement of this structure was modest, The PI has been implicated in somatosensory processing and as a food reward region (82–84). Pronounced PI activity has positive associations with subjective cravings elicited by images of addictive stimuli (73), imagination of taste and smell during visual food cue presentations (85, 86), passive visualization of food stimuli (87), consumption of highly palatable substances (88), receipt of a preferred food/drink odors (86), internal hunger state (89), and gastric distention in the absence of actual food intake (90). In line with such data, images of Chinese foods may have elicited stronger recollections of taste properties (e.g., intensity, valence and identity of taste) and corresponding increases in PI activity compared to Western food images (91). Localization to the right PI also aligns somewhat with early support for right hemisphere dominance found for taste-related insula activation (92).

Notwithstanding the need for replications, these activation findings provide initial support for the hypothesis that, among Chinese participants, Chinese (versus Western) food images elicit stronger automatic responses in brain regions related to reward, attention, food intake and taste perception. Conversely, however, there were no activation differences in other reward circuit regions such as the nucleus accumbens, putamen, caudate, OFC, anterior insula, or amygdala (21, 23–25, 93). The absence of differences in ratings of liking, wanting, and difficulty resisting depicted Chinese versus Western food images may have contributed to the small number of reward area differences between these image sets. In addition, the use of Chinese versus Western food image sets matched for overall calories, familiarity, nutritional content, and flavors may have attenuated the number of differences in reward region responsiveness.

This explanation is not entirely sufficient, however, because supplementary analyses (Supplementary Table 1) revealed very few food versus furniture image differences in reward area activity compared to activation of regions involved in visual processing and attention. Research design features may have contributed to the pattern of food versus furniture activation effects. Specifically, participants did not undergo the study in a food deprived state because effects of culture, rather than hunger, were the central focus. In addition, images were presented during a passive viewing task rather than in the context of active engagement instructions (imagined taste versus imagined use). As such, it is possible that the sight of food (versus furniture) did not produce activation differences in associated brain regions (e.g., OFC) having links with reward because hunger and taste associations were attenuated (66). Future extensions evaluating activation differences in indigenous versus non-indigenous food image contrasts as well as contrasts between these food image types and non-food images under conditions of increased hunger (e.g., following an overnight fast) may provide additional insights about the role of culture as an influence on neural responses to food images.

Other activation differences were observed in regions related to vision and attention. For example, Chinese foods elicited stronger responses in middle and inferior occipital gyri. In general, these regions are involved in visual processing, yet meta-analytic evidence has linked elevations to preferred, “highly hedonic” foods relative to not-preferred, “bland/neutral” foods (93). It is not clear that our findings for these areas align with interpretations of non-conscious or conscious preferences for depictions of more hedonic foods because subjective evaluations of Chinese versus Western food images did not differ. Re-evaluations in the context of paradigms involving forced choice preferences for Chinese versus Western food items might clarify the validity of such interpretations.

Finally, Western food images elicited comparatively stronger activity in visual association regions (i.e., ITG, left middle occipital gyrus, fusiform gyrus) linked to complex visual tasks including attention to shapes, visual processing of objects and color, visual form discrimination, object recognition and object identity retrieval (94). Given that Chinese samples are more likely to eat Chinese foods than Western foods (1, 14, 15, 17), enhanced activity in these regions might reflect lower consumption rates of at least some depicted Western foods. However, extensions that include consumption frequency (e.g., food diaries) are needed to test this conjecture.

### Subjective evaluations of Chinese versus Western food images

No Chinese versus Western foods image differences in liking, wanting, and difficulty resisting were reported, contrary to expectations. These null effects may have been due to equating food image sets for calorie levels, content, familiarity, and flavor characteristics. Furthermore, self-report judgments of Chinese versus Western food images may be an imprecise proxy for actual eating patterns or preferences in real world situations. Conversely, these null effects may have reflected increases in Western food outlets and gradual shift toward more Westernized diets in Chinese samples (2, 18). Relatedly, because our sample comprised young adults, differences in subjective evaluations of depicted Chinese versus Western foods may have been attenuated because neophobic attitudes toward Western food are strongest among older Chinese adults (1).

### Associations of subjective evaluations with identified regions of interest

Aside from showing differential responsiveness during exposure to Chinese (versus Western) food images, the cluster comprising the STG, SMG, MTG, IPL, and PI also had significant positive correlations with wanting and difficulty resisting ratings of depicted food images following the scans; these correlations were stronger in relation to ratings of Chinese food images though associations with Western food image ratings were also statistically significant. Despite the lag between viewing and rating the food images, these correlations appear to be consistent with evidence of functions that include taste perception (34, 72), cravings (66, 79), reward processing and inhibitory control (59, 75, 88), and attention (26, 49). While few past studies have assessed “difficulty resisting” food items or “dietary restraint” (49, 95), such research also taps evaluations of whether or not to consume depicted foods. The underlying neural system comprises the dorsolateral, inferolateral and superior prefrontal cortex, dorsal anterior cingulate cortex, basal ganglia nuclei, and cerebellum involved in attention, self-regulation and inhibitory control (49). Although preliminary, our results implicate possible involvement of a cluster comprising the right STG, SMG, MTG, IPL and PI in wanting and reported difficulty resisting visual food cues, though significantly different effect size strengths in responsiveness were not elicited by Chinese versus Western food images.

### General implications for future research

Our main findings have potentially important implications for research and practice. Given that Chinese (versus Western) food images elicited significant neural activation differences in select clusters having involvement in gustation, cravings, reward processing and inhibitory control, attention, and visual processing, the implicit assumption that Western food images elicit neural responses that are universally representative of all cultural groups is not well founded. From this perspective, researchers should be cautioned against naively using “standardized” image sets of Western foods within neuroimaging studies conducted in China or other non-Western countries that have their own culinary traditions and preferences due to possible threats such images might pose for ecological validity or relevance. That is, neural responses based on exposure to images of Western foods may not always reflect typical neural responses to depictions of culturally indigenous foods that have more potential salience for actual diets of non-Western groups.

That said, the limited number of activation differences between Chinese versus Western food images and the absence of conscious subjective liking, wanting, and difficulty resisting food images from the two cultural contexts suggest that Western food products have become increasingly familiar and more popular in China, at least among young adults (2, 18). As such, hybrid sets of Chinese and Western food images may have more ecological validity than Chinese food images alone do within particular population groups. More broadly, the findings underscore the importance of considering the appropriateness of indigenous and/or standard (Western) food images in designing ecologically valid neuroimaging studies of visual food cues in non-Western groups.

In relation to possible applied implications, food stimuli can trigger significant neural, physiological and psychological changes (96). Therefore, food images can be used in research and clinical practice to detect and treat disordered eating behaviors. In light of their increased ecological validity in this research, Chinese food images from this study may aid in developing culturally informed assessments for detecting eating disturbances and stimuli for interventions (e.g., exposure therapy, imagery) designed to reduce these problems among people currently struggling with these problems within Chinese cultural contexts.

### Limitations and future directions

Despite its novel focus and potential implications, the main limitations of this study should be acknowledged. First, it is not clear whether findings generalize to response difference comparisons of indigenous versus foreign food image in other non-Western cultural contexts or other age groups. In light of evidence that older Chinese adults are more prone to neophobia of Western foods (1), it is possible that both subjective preferences and neural responses of older respondents are even more distinct from those observed in our sample. Extensions should also be conducted within other age groups and samples from other cultures. Second, although food image sets were equated for calorie levels, content, familiarity, and flavor characteristics, it is possible that prior Chinese versus Western food consumption patterns reflected or affected associated preferences and neural responses. We did not assess participants’ recent diets because strategies such as weekly food diaries are not well-validated in Chinese samples and are susceptible to potential biases related to a reliance upon retrospective recall. Nonetheless, as such procedures become more refined, effects of individual differences in indigenous versus non-indigenous food consumption patterns can be evaluated. Finally, because participants served as their own controls within this study, activation differences observed in this study could not be explained by numerous individual differences influences. Regardless, future studies might consider moderating effects of individual differences in biological factors (e.g., menstrual cycle stage for women, blood glucose levels), psychological influences (e.g., food neophobia, reward sensitivity), and study design features (e.g., deprivation versus satiation) on conscious reactions and neural responses to indigenous food versus Western food images.

## Conclusion

To our knowledge, this study is the first to examine subjective reactions and neural responses to images of indigenous versus non-indigenous foods. Analyses indicated that even though conscious preferences did not differ between Chinese versus Western depictions of food, Chinese food images elicited comparatively stronger activation in particular regions linked to reward, higher-order cognitive control (i.e., cerebellum crus, superior temporal gyrus inferior parietal lobule, posterior insula, occipital gyrus) as well as weaker activation in regions related to visual object recognition (ITG, occipital gyrus) and processing (cuneus). Results are preliminary but underscored culture as a potentially potent influence on neural responses to visual food cues and raised possible external validity concerns with non-critical use of Western food images in studies of non-Western samples. Consequently, in designing culturally informed research or interventions that involve visual depictions of food in non-Western contexts or distinct cultural groups in Western countries, the salience of indigenous food images versus “standard” Western food images warrants consideration.

## Data availability statement

The raw data supporting the conclusions of this article will be made available by the authors, without undue reservation.

## Ethics statement

The studies involving human participants were reviewed and approved by Ethics Committee of Southwest University. The patients/participants provided their written informed consent to participate in this study.

## Author contributions

TJ and JP: conceptualization. TJ, JP, and XX: methodology and resources. XX: software, investigation, and data curation. XX and TJ: validation, formal analysis, writing—original draft preparation, and visualization. TJ, XX, and AS: writing—review and editing. TJ: supervision, project administration, and funding acquisition. All author read and agreed to the published version of the manuscript.
