# Supplementary material for: Neural responsiveness to Chinese versus Western food images: An functional magnetic resonance imaging study of Chinese young adults
Source: Front Nutr. 2022 Aug 12;9:948039. doi: 10.3389/fnut.2022.948039 (PMC9411937; doi:10.3389/fnut.2022.948039)
Supplement: Supplementary file 1 [file Data_Sheet_1.PDF]

Supplementary Material

1 Supplementary analyses of neural activation differences between food versus furniture image categories

Supplementary Table 1 summarizes activation results based on contrasts of Chinese (or Western) food images with furniture images. Chinese food images elicited comparatively stronger activation in the left middle occipital gyrus, calcarine, and bilateral lingual gyrus and comparatively weaker activity in the right calcarine, left precuneus and cerebellum. Western food images elicited relatively increased responsivity in the lingual gyrus, cerebellum, and superior orbital frontal gyrus, and comparatively attenuated activity in the fusiform gyrus, precuneus, calcarine, cerebellum, frontal pole, middle frontal gyrus and angular gyrus (Supplementary Table 1, Supplementary Figure 1).

Supplementary Table 1.

Sample activation differences for contrasts of Chinese and Western food images with furniture images (N = 66).

Contrast	Anatomical Label	BA	Hem	Voxels	x	y	z	Peak T-value
ChFd > Furn	Lingual gyrus	18	R	53050	24	-96	-7	21.25
	Lingual gyrus	18	L		-20	-98	-14	20.66
	MOG	11	L		-24	34	-16	10.79
	Calcarine	30	L	274	-6	-54	9	5.69
ChFd < Furn	Calcarine	18	R	53050	20	-86	14	9.37
	Cerebellum	18	L		-10	-78	-9	8.84
	Precuneus	7	L		8	-78	51	8.78
WeFd > Furn	Lingual gyrus	18	L	37260	-20	-98	-14	18.55
	Lingual gyrus	18	R		24	-96	-7	17.81
	Cerebellum	19	L		-34	-74	-18	11.47
	Superior OFG	11	R	298	22	32	-21	9.46
WeFd < Furn	FFG	37	L	37260	-30	-44	-12	10.94
	Precuneus	7	R		10	-80	48	9.06
	Calcarine	19	R		22	-86	18	8.65
	FFG	37	R		30	-40	-14	10.16
	Cerebellum	18	R	470	12	-72	-9.2	6.91
	Frontal pole	8	L	378	-24	6	53	5.81
	Frontal pole	46	L		-42	56	4.6	5.52
		47	L	350	-50	44	-14	4.76
	Angular gyrus	39	L	468	-38	-48	32	5.39
	MFG	8	R	290	24	10	44	4.47

Notes: BA: Brodmann area; Hem: Hemisphere; x, y, z coordinates in MNI space (Montreal Neurological Institute); ChFd: Chinese food; WeFd: Western food; Furn: Furniture; L and R: left and right; Reported brain activation was significant at corrected $p < 0.05$; MOG: middle occipital gyrus; OFG: orbital frontal gyrus; MFG: middle frontal gyrus; FFG: fusiform gyrus.

Supplementary Figure 1.

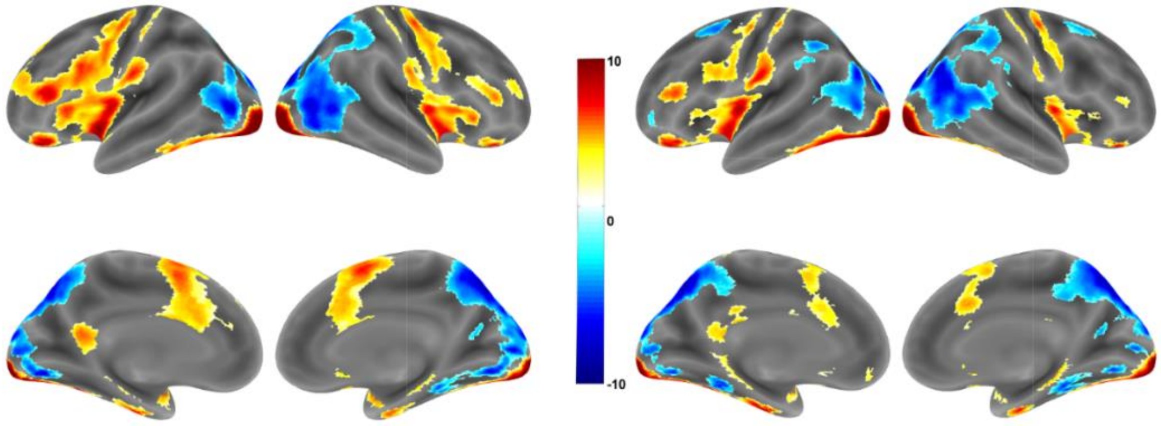

Supplementary Figure 1. Brain regions activated by the two conditions (left: Chinese food images, right: Western food images) relative to the non-food images (furniture) (corrected $p < .05$). Warm tones indicate greater activation during the food condition and cool tones indicate greater activation during the non-food condition.
